# ‘How do you sleep at night knowing all this?’: climate breakdown, sleep, and extractive capitalism in contemporary literature and culture

**DOI:** 10.1080/0950236X.2023.2265887

**Published:** 2023-10-04

**Authors:** Diletta De Cristofaro

**Affiliations:** Humanities Department, Northumbria University, Newcastle upon Tyne, UK

**Keywords:** Contemporary literature, sleep, climate breakdown, sleep crisis, extractive capitalism

## Abstract

Contributing to the emerging field of critical sleep studies, and developing an intervention situated at the intersection of the environmental and the medical humanities, this article considers a range of contemporary texts: Jenny Offill’s realist novel *Weather* (2020), Karen Russell’s *Sleep Donation* (2014), Cherie Dimaline’s *The Marrow Thieves* (2017) and *Hunting by Stars* (2021) – three examples of the ‘sleep-apocalypse’ genre – Finegan Kruckemeyer’s play *Hibernation* (2021), and the Perfect Sleep app by Tega Brain and Sam Lavigne (2021). I show how these texts do not just simply reflect the negative effects that climate change has on sleep health, which are manifold, as scientific research evidences. Rather, cultural production arguably draws attention to structural parallels between the climate crisis and the so-called sleep crisis, namely, contemporary society’s presumed widespread sleep deprivation and rise in sleep disorders. Both crises are the product of a capitalist system geared towards continuous extraction – and exhaustion – of resources, from the Earth and human bodies. Thus, in the texts considered, sleep is explored, on the one hand, as a casualty of the climate crisis and, on the other hand, as something whose value we need to reassess as part of our ongoing work to avert climate collapse.

In the Summer of 2022, the Northern hemisphere experienced unprecedented high temperatures, with heatwaves striking many parts of Europe, the US, and China.^1^ Human-caused climate change was behind these extreme temperatures, with many pointing out that heatwaves are only likely to get worse in years to come.^2^ In the UK, a country still relatively unaccustomed to long stretches of high temperatures in the Summer, the media discourse about how to cope with the record-breaking temperatures often focused on sleep, and how-to articles providing top tips for a good night sleep in the heat proliferated.^3^ This fixation on sleep might seem odd. Yet scientific studies looking at the health impacts of climate change are increasingly turning their attention to sleep. As explored in what follows, sleep also frequently appears in contemporary literary and cultural engagements with the prospect of climate breakdown. This article breaks new ground using media and scientific discourse on sleep and climate change as a lens of enquiry for approaching a range of contemporary texts, from realist novels like Jenny Offill’s *Weather* (2020) to ‘sleep-apocalypse’ fictions like Karen Russell’s *Sleep Donation* (2014) and Cherie Dima-line’s *The Marrow Thieves* (2017) and *Hunting by Stars* (2021), from plays like Finegan Kruckemeyer’s *Hibernation* (2021) to digital culture like the Perfect Sleep app by Tega Brain and Sam Lavigne (2021).

Contributing to the emerging field of critical sleep studies, and developing an intervention situated at the intersection of the environmental and the medical humanities, the present article shows how these texts do not just simply reflect the negative effects that climate change has on sleep health, which are manifold, as scientific research evidences. Rather, cultural production arguably draws attention to structural parallels between the climate crisis and the so-called sleep crisis, namely, contemporary society’s presumed widespread sleep deprivation and rise in sleep disorders. Both crises are the product of a necrogenic capitalist system geared towards continuous extraction – and exhaustion – of resources, from the Earth and human bodies.^4^ Thus, in the texts considered in the following pages, sleep, and more broadly rest, are explored, on the one hand, as a casualty of the climate crisis, specifically, of the extractive capitalism at the crisis’ heart, and, on the other hand, as something whose value we need to reassess as part of our ongoing work to avert climate collapse.

## Intersections of the climate and sleep crises

Climate change has been identified as the ‘biggest global-health threat of the twenty-first century’, linked as it is to deadly heatwaves and extreme climatic events, changing patterns of diseases, issues of access to food and clean water, and air pollution.^5^ Recent studies have added sleep to these health risks: since human sleep is regulated by temperature, climate change-induced increases in nighttime temperatures are leading to rising rates of sleep deficiency.^6^ This is alarming as sleep deficiency has disastrous consequences on health, such as lower immune function and higher risks of developing heart disease, diabetes, depression, and even some types of cancer.^7^

Mental health is also severely affected by the climate crisis, with research showing both the crisis’ direct effects – namely, mental health conditions, such as PTSD, anxiety or depression, developing in those that have experienced climate disasters – and its indirect effects,^8^ such as the profound implications for mental wellbeing of witnessing other people’s lives being devastated by these disasters and fearing for the future. Eco-anxiety, a term that ‘refers to the chronic fear of environmental doom’, is indeed diffuse.^9^ And, as the link between poor mental health and poor sleep is well-documented,^10^ it is hardly surprising that increased levels of climate change-induced anxiety, stress, depression, and trauma are leading to rising rates of sleep disorders.^11^

This sense of sleep being under threat because of the effects of the climate crisis intersects with a wider sense of a sleep crisis in contemporary society, evident in a proliferation of pronouncements in scientific analyses, the media, and everyday life conversations, as well as of cultural images and representations, articulating the contemporary moment as one defined by the steep decline in sleep quality and quantity and the equally steep increase of the number of people suffering from sleep disorders. I refer to this proliferation as ‘sleep crisis discourse’. There is less scientific consensus about the sleep crisis than there is about the climate crisis. According to some studies, ‘people are getting one to two hours less [sleep] each night than their ancestors did 50-100 years ago’ and ‘sleep pathologies are approaching epidemic levels’.^12^ While most these studies – and indeed, typically, the sleep crisis discourse itself – focus on the Global North, ‘evidence is emerging that sleep duration and quality are even lower in developing countries and among the poor in rich countries’.^13^ The reasons behind this crisis include ‘the culture of long work hours, more shift-work, long commutes, global communication across multiple time zones, and… the 24-hour availability of almost everything’.^14^ Other scholars, though, contest the ‘evidence that, today, we sleep fewer hours (maybe a fewer minutes but not hours) than we did then’ and suggest that the sleep crisis simply makes for a good media narrative.^15^ Media pieces reporting society’s presumed sleep crisis – and what to do about it – have indeed proliferated in recent years and have been given new impetus by the COVID crisis.^16^

Yet, beyond the media, and irrespective of whether twenty-first-century society is in the grip of a sleep-related public health emergency or not, the discourse of the sleep crisis is widespread in contemporary culture. As discussed in this article and elsewhere,^17^ we find this discourse in fiction, non-fiction (from insomnia memoirs to self-help books), as well as digital and visual culture. Concerns about a sleep crisis and diffuse exhaustion are not necessarily new.^18^ But ‘The rhetoric of our age is unique in that anxieties about exhaustion, sustainability, and resilience no longer concern only the mind, body, or society but our very habitat’.^19^ Arguably, mirroring extractive capitalism’s exhaustion of the biosphere, one of the dominant affects of this system is exhaustion,^20^ which explains widespread perceptions of the contemporary moment as the site of a sleep crisis. Indeed, by exploring a real crisis – climate breakdown – through a crisis that may not be genuine but is, nevertheless, strongly felt – the sleep crisis – the texts considered in this article convey the affective imports of the former crisis.

My work on the literature and culture of the sleep crisis is situated within critical sleep studies, a developing ‘subfield of humanities and social sciences [that examines] the sociocultural meanings of sleep’.^21^ Since sleep has always been ‘a cipher, metaphor, or vehicle, call it what you will, for something else’,^22^ I argue that the discourse of the sleep crisis – be this crisis real or just a matter of perception – serves as a prism through which culture refracts a series of anxieties about contemporary life.^23^ In this article, in particular, I show how the climate and the sleep crises coalesce in the texts discussed to articulate reflections on the unsustainability of the current capitalist system, for the environment and people’s lives.

This examination of the relationship between the sense of a sleep crisis and extractive capitalism builds upon a core research area for critical sleep studies, which explore how changes in capitalism inform changes in sleep.^24^ In the present article, I make two new contributions to the field: firstly, through my engagement with literature, and specifically very recent literature, which remains a minor focus for critical sleep studies (albeit interest in this area is growing).^25^ Secondly, I wish to develop what I see as one of the most promising, yet still only emerging, directions in the field,^26^ namely, the theorisation of the relationship between conceptions and understandings of sleep and conceptions and understandings of nature and the environment, and therefore contribute to the theorisation of the relationship between the politics of sleep and the politics of the environment in the twenty-first century. In this sense, my article also seeks to respond to growing calls for more sustained exchanges between the environmental and the medical humanities,^27^ for sleep is key to both human and planetary health.

## Eco-anxiety in Jenny Offill’s *Weather*

Written in Jenny Offill’s signature fragmentary style, *Weather’s* first-person narrative ceaselessly moves between scenes from the daily life of the protagonist, Lizzie, her internal monologues, snippets of conversations, questionnaires, quotations, and emails – a narrative mode that leaves the reader in charge of discerning meaningful patterns and connections between the fragments. The novel’s plot is minimal and mostly propelled by a diffuse sense of existential dread that encompasses the crisis of American democracy – *Weather* is set around the 2016 election of Donald Trump, although the former US President is never explicitly named – and the more global crisis to which the title alludes, the climate crisis. Indeed, *Weather* has been defined by critics as a ‘climate anxiety novel’ that ‘transform[s] the novel of consciousness into a record of climate grief.^28^ One of the ways *Weather* registers this eco-anxiety and grief is, arguably, Lizzie’s insomnia.

Lizzie works as a librarian but a few pages into *Weather* she begins supplementing her family’s income by helping her former graduate advisor, Sylvia, answering emails about her podcast *Hell and High Water*. This programme, as the apocalyptic-sounding title suggests, is about the ‘invisible horsemen galloping towards us’, first and foremost environmental break-down.^29^ Working for Sylvia, Lizzie learns that climate scientists are ‘in a state of barely suppressed panic’, as ‘Everything is happening much faster than expected’.^30^ ‘How do you sleep at night knowing all this?’, Lizzie asks Sylvia.^31^ Sylvia’s sleep does not appear to be affected: as she explains, she has ‘known it for a long, long time’ though, as Lizzie notes, Sylvia’s ecoanxiety still manifests in other ways, including her ‘going, going, gone trips’, where she visits places that are disappearing because of the climate emergency.^32^ Lizzie’s sleep does suffer, however, her eco-anxiety compounded by the fear for the future of her child Eli.

One bedtime, she becomes particularly concerned with climate departure, namely, the point at which the climate will cease resembling that of the past.^33^ ‘According to the current trajectory’, Lizzie knows that the climate departure for New York, where her family lives, is 2047, when the city ‘will begin to experience dramatic, life-altering temperatures’.^34^ Thus, she starts staying up later and later into the night googling preppers’ survival tips and endlessly ruminating the question she ‘can’t seem to escape… What will be the safest place’ for Eli?^35^ Her anxious nighttime ruminations are made all the more poignant by the fact that Lizzie knows that a definite answer to this question is impossible. *Weather* returns time and again to the desire for a safe haven from climate breakdown only to stress that it is just wishful thinking. As a scientist puts it, ‘I don’t think there will be any safe places…. the impacts are going to be big’.^36^ The nefarious effects of eco-anxiety on Lizzie’s sleep take hold at the hopeless intersection of desperately wanting to prepare her family for the climate emergency and fearing that no preparedness is truly possible: ‘I know … all my preparations for the apocalypse are doomed. I will die early and ignobly’, she muses.^37^

*Weather* has been criticised for failing to coordinate ‘individual singularity with a collective social imaginary’.^38^ The novel’s issue, Rithika Ramamurthy claims, is that it recasts the environmental crisis as ‘private emotional torment’, giving us only ‘one person’s perception of how bad things make her feel’.^39^ While, as a novel of consciousness, *Weather* is inevitably focused on the individual scale, and specifically, on the limited perspective of an individual from the Global North, I would argue that Offill does also make gestures towards the ‘totality of social relations in the extraction era’, as climate fiction should do.^40^ Other critics have pointed out that the novel’s image of the mesh – an image that evokes Timothy Morton’s homonymous concept identifying ‘the interconnectedness of all living and non-living things’^41^ – is central to this project:

In one of Sylvia’s podcast episodes, an ecologist uses the word ‘mesh’ to describe the interdependence of all lifeforms on earth. The word mesh recalls Lizzie’s emotional ‘enmeshment’ with her dysfunctional adult brother. In sewing this precise ‘little pattern’, Offill troubles the distinction between the ecological and the personal, the big and the small, the important and the trivial.^42^

In fact, ‘The form of the book enacts that very enmeshment – the fragments that hook into each other, rhyme and repeat’.^43^ I suggest that sleep is another theme through which *Weather* mediates between the individual and the systemic, a mediation that must feature in climate fiction if this is to engage with the scale of the current environmental crisis, as well as its causes and solutions.

As we shall see throughout this article, sleep is ideally positioned to articulate this mediation. Typically, sleep is conceptualised as a time when the individual withdraws from the world. And yet, sleep is a phenomenon that is deeply historical and informed by society for, as suggested by Lizzie’s ecoanxiety, how we sleep or do not sleep is bound up with the world in which we live. Sleep, Simon J. Williams writes, ‘is political through and through’.^44^ Indeed, just like the environment itself, sleep is arguably a site of what Nancy Fraser identifies as boundary struggles. Within capitalist societies, boundary struggles are ‘nodes of contradiction and potential crisis’ that emerge ‘at the points where production meets reproduction, economy meets polity, and human society meets non-human nature’.^45^ Sleep is crucial to social reproduction, namely the processes that reproduce society and ensure its functioning, as, put simply, we need sleep to live *and* work. But what the sleep crisis discourse registers is a sense that capital is increasingly eroding the time for sleep and rest, producing widespread exhaustion.

In *Weather*, sleeplessness does not just feature as a result of Lizzie’s individual eco-anxiety but also, qua sleep crisis, as the product of the same capitalist system responsible for the environmental crisis. ‘Everyone I know’, Lizzie muses, ‘is trying to sleep less. Insomnia as a badge of honor’.^46^ Within a system aimed at extracting the most value and profit from both the natural world and human labour, people are pushed to constantly compete with each other, a ‘philosophy of late capitalism’ that Lizzie summarises through the parable of two hikers chased by a hungry bear: ‘You can’t outrun a bear’, one of the hikers whispers to the other, as he sees his companion putting on his running shoes; ‘I just have to outrun you’, the other responds.^47^ This competition, in turn, engenders the sense that people should curtail sleep in order to achieve more, or even simply to survive; a ‘dominant sleep-negative agenda’ that characterises contemporary work culture and the 24/7 society.^48^ Lizzie’s taxi driver, for instance, ‘sleeps at work …so as to never miss a call’, in a desperate bid to save his failing business.^49^ But ‘There’s a reason [sleep deprivation is] used as a tool of torture’, Lizzie cautions us, evoking sleeplessness’s disastrous impacts on physical and mental health.^50^

Yet, sleeplessness, as ‘the state in which producing, consuming, and discarding occur without pause’, is not just ‘hastening the exhaustion of life [but also] the depletion of resources’, for ‘24/7 is inseparable from environmental catastrophe’.^51^ That is why, as we shall see further in the article’s concluding section, sleep and rest can be conceived as tools of resistance against the systems that produce this exhaustion of resources, both bodily and planetary. To return to Fraser, boundary struggles are both sites of crisis and ‘stakes of struggle’.^52^ Sleep, in other words, is not just threatened by extractive capitalism and the 24/7 ideal of continuous operation, as the discourse of the sleep crisis – be this real or perceived – suggests, but it is one of the ‘thresholds at which society could defend or protect itself’.^53^ Hardly by chance, *Weather’s* concluding ‘obligatory note of hope’ – a term first used by Sylvia when she tells Lizzie that she feels she has to include a hopeful note in all her work to incite action in the face of the climate crisis^54^ – is not just the eponymous website whose link appears on the page before the acknowledgments and which is the novel’s digital appendix^55^ but the narrative’s final restorative scene in bed. Lizzie falls asleep in a moment of peaceful familial comfort, while her husband arranges her blanket for warmth and their dog is at the foot of the bed.^56^

Through Lizzie’s insomnia, the manifestation of her eco-anxiety, *Weather* captures the nefarious effects of the climate emergency on sleep health. And, by mediating between individual and systemic sleeplessness, the novel begins to gesture to structural parallels between the environmental crisis and the sleep crisis, parallels that are more fully articulated in the next set of texts discussed.

## Sleep-apocalypses in Karen Russell’s *Sleep Donation* and Cherie Dimaline’s *The Marrow Thieves* and *Hunting by Stars*

The genre par excellence of the sleep crisis is what I would call sleep-apocalypse fiction. In these texts, anxieties about contemporary society’s sleep encapsulated by the discourse of the sleep crisis are blown up into fully apocalyptic proportions through catastrophic pandemics of sleep disorders, mostly – albeit not exclusively – insomnia.^57^ Typically, the causes of the sleep-apocalypses remain nebulous, though fictional speculations revolve around the same causes posited to underlie the sleep crisis of our own society, from 24/7 capitalism to the relationship with electronic devices that keep people up well into the night. This is not to say that sleep-apocalypse fiction unquestioningly supports the hypothesis of a contemporary sleep crisis. Rather, these texts invite us to consider the anxieties behind this discourse and the values ascribed to sleep in today’s world. Drawing parallels between their sleep-apocalypses and the climate crisis, bodily and planetary exhaustion, the novels considered in this section – *Sleep Donation*, *The Marrow Thieves* and *Hunting by Stars* – articulate sustained critiques of extractive capitalism by interrogating sleep as another resource to exploit and exhaust for capital.

Karen Russell’s *Sleep Donation*, originally published in 2014 in a digital-only format and re-issued in print in 2020, imagines a world very similar to our own, except for a pandemic of total sleep loss – terminal insomnia – which leaves a significant portion of the US population, and later of the entire planet, progressively unable to sleep until these people die from constant vigilance. This emergency is more than once called sleep crisis in the novel and, echoing the claims of the sleep crisis discourse that contemporary society is chronically sleep deprived, the new disorder is initially dismissed ‘as an exaggeration of a universal American condition. Who was sleeping enough? Nobody!’.^58^ Soon, however, scientists realise the severity of the situation and begin researching what may have caused the development of terminal insomnia. This proves to be a million-dollar question, with speculations ranging from ‘the oceans’ tides, magnetism, the poles, the hemispheres, the net of light and shadow on the globe’ to ‘our twenty-four-hour news cycle, our polluted skies and crops and waterways, the bald eyeballs of our glowing devices’.^59^ The tone in which Trish – *Sleep Donation*’s protagonist and first-person narrator – reports these ‘frantic lists of increasingly wild and impossible causes [is] parodic of the language of crisis, which presents fears about broader social phenomena as acute public health catastrophes’.^60^ Yet, while humorously suggesting that any claim of a sleep crisis in today’s world should be taken with caution, *Sleep Donation* also addresses a more fundamental question: why is this discourse so popular? Ultimately, the novel takes seriously the hypothesis that ‘[we] are sitting in an electric chair that we engineered’,^61^ namely, that twenty-first-century systems and infrastructures, including those responsible for the environmental crisis (notice the reference to pollution above), are to blame for the novel’s sleep crisis and, by extension, for today’s diffuse sense of exhaustion.

In an interview, Russell states that her fictions’ key purpose is to ‘restore the real horror of how our economy runs to us’.^62^ *Sleep Donation*’s sleep crisis arguably serves this function by drawing attention to extractive capitalism’s insatiable commodification and plundering of the human and more-than-human world, processes that are leaving people exhausted and driving the planet to the brink of climate breakdown. Tellingly, Russell’s descriptions of the sleep crisis are couched in a language that evokes the environmental crisis. Scientists ‘predict “a global *desertification* of dreams”. Soon, …. Sleep will go *extinct*. And eventually,.. so will we’.^63^ The approaching end of sleep is poignantly depicted as humankind’s terminal divorce from the more-than-human world: scientists maintain that the suprachiasmatic nucleus, which regulates the human circadian rhythms, ‘the master clock that syncs us to one another, and to the earth’s rotation … [and] To all the sister kingdoms’, will soon stop for everybody, signalling our species’ end.^64^ This divorce is nothing else than the logical endpoint of humankind’s all-too-often instrumental relationship with nature, a relationship that catastrophically fails to acknowledge how dependent humans are on the natural world.

As a bodily function, sleep is arguably aligned with nature in the West’s imagination, occupying what Val Plumwood identifies as ‘the underside of rationalist dualisms that oppose reason to nature, mind to body, emotional female to rational male, human to animal, and so on’; in other words, the side of these dualisms that needs to be dominated and controlled.^65^ That is why the history of sleep is, at its heart, the history of how it has been manipulated to support the development of capital, just as nature has.^66^ In the contemporary moment, the sleep crisis discourse registers anxieties about how 24/7 capitalism ‘renders plausible, even normal, the idea of working without pause, without limits’,^67^ an ideal of continuous operation that pushes people to curtail the time for rest and is, we have seen, imbricated with the environmental crisis. *Sleep Donation*’s terminal insomnia, as well as the eponymous sleep donations, the condition’s only treatment, give these anxieties a tangible shape, pointing to parallels between how sleep and nature are valued, or in fact devalued, under extractive capitalism.

Sleep donations too are described in ways that are reminiscent of the climate emergency and, specifically, of the indiscriminate extraction of fossil fuels and natural resources. Sleep donations are a form of sleep-transfusions from healthy sleepers to the sick. However, there are not enough donors to eradicate the pandemic. Furthermore, the transfusions are at high risk of being compromised by incredibly contagious nightmares, which generate a second – and equally disastrous – sleep-pandemic, this time of ‘elective insomnia’, as people do all they can to avoid sleeping and being haunted by these nightmares. That is why the most sought-after donors are babies, in particular ‘Baby A’, whose sleep is the purest and not yet contaminated by any nightmares. Sleep donations thus come to symbolise how the prospect of climate breakdown is ‘syphoning off the dreams of children’, together with their future.^68^ Promising Baby A’s mother that they will not take more sleep than it is safe from her daughter, Trish – who works for the seemingly non-profit organisation that administers the sleep draws and donations, the Slumber Corps – quickly realises the utter untenability of her assurances by evoking the spectre of resource-exhaustion:

I make this promise at a moment when people are plunging their straws into any available centimeter of shale and water, every crude oil and uranium and mineral well on Earth, with an indiscriminate and borderless appetite…. According to … estimates, our species will be extinct in another four generations, having exhausted every store of water and fuel on the planet.^69^

Can she really promise they will respect Baby A’s limits, Trish wonders, when ‘We have never in our species’ history respected Nature’s limits’?^70^ It is no coincidence that the media call sleep donations ‘mining’,^71^ further emphasising the structural parallels between the insatiable extraction of sleep from donors and of resources from the Earth – extractive processes that conveniently ignore these resources’ finitude.^72^

In *Sleep Donation*, sleep is the latest frontier of capitalist extraction and exhaustion, which, in turn, invites us to consider the extent to which this might already be true beyond the novel. When we instrumentally think of sleep as something that will make us more productive during the day, rather than as time that is valuable in and of itself, or even time that is valuable also because it is outside the time of production, we arguably fall prey to a logic whereby sleep is effectively a resource for extractive capitalism. Consider that even the ‘sleep-positive agenda’ itself,^73^ which seeks to champion the importance of sleep against the supposed sleep crisis, often does so by highlighting the economic costs of insufficient sleep and its nefarious effects on productivity.^74^

*Sleep Donation*’s plot develops as Trish finds out that the Slumber Corps is not a benevolent non-profit organisation but is instead behind a global black market of sleep, a scenario straight out of the playbook of disaster capitalism, which turns every crisis into an opportunity for profit. Sleep, at least for now, ‘collide[s] with the demands of a 24/7 universe … [and] subsists as one of the great human affronts to the voraciousness of contemporary capitalism’ through ‘the incalculable losses it causes in production time, circulation, and consumption’.^75^ But *Sleep Donation* imagines the fall of sleep as the last bastion against capital’s advance. Sleep is an incredibly scarce, and therefore extremely valuable, commodity in the novel. What is more, since sleep donations are not actually donations but, rather, extractions that produce profit, they are, in fact, labour. Work takes over every aspect of people’s lives, including the night-time, which typically represents a pause – limited as this may be – in our participation in the economy and its nefarious environmental impacts.

The sleepless, as Deckard notes, are ‘grotesque parodies of capitalism’s fantasy of a productivity fix in the form of unsleeping workers, [in that] far from enabling perpetual productivity, their wakefulness causes progressive debilitation’.^76^ But those who donate their sleep are the realisation of this fantasy, which only furthers extractive capitalism’s plundering of the human and more-than-human world. Jeremy, one of Trish’s colleagues, is the perfect example: not only does he work the equivalent of three jobs at a very low salary but he also compounds this obscene amount of work hours with sleep donations (namely, more labour). ‘Since the crisis began, Jeremy’s given half a year of his life: 4,392 h – he grins proudly – which is far in excess of the legal limits’.^77^ Donating beyond these limits, however, has ‘the same consequences of sleep loss that afflict [the terminal] insomniacs’, in a word, exhaustion. In the figure of Jeremy, ‘stumbling around … like a zombie …., zonked from a nine-hour draw’^78^ we see the terrifying costs of the capitalist fantasy of a 24/7 extraction of profit from human labour, and therefore, a more accurate manifestation of the anxieties behind the sleep crisis discourse than the terminal insomniacs.

*The Marrow Thieves* and its sequel *Hunting by Stars*, written by Métis author Cherie Dimaline, add an important element to *Sleep Donation*’s critique of extractive capitalism via sleep, namely, the enduring legacies of colonialism on this system and on the health and wellbeing of Indigenous people. Dimaline’s imagined future has been devastated by two ongoing disasters: climate breakdown and a sleep-apocalypse that takes the form of a pandemic of dreamlessness. This pandemic only affects the settler population of North America; Indigenous people can still dream and are therefore hunted down and ‘harvested’ to develop treatments. Settlers believe that the ability to dream is stored in Indigenous peoples’ bone marrow, which is forcibly extracted with deadly consequences in institutions that constitute the return of the Canadian Residential Schools, a system that was aimed at eradicating Indigenous culture and knowledge. This return is one of the strategies Dimaline deploys to link her environmentally-devasted future to the colonial past – a past that, the novels imply, is not really past at all – thus situating extractive capitalism as part of a long history of brutal theft of Indigenous lands, labour, and lives.

Dimaline’s sleep-apocalypse is the consequence of climate breakdown. ‘Earthquakes in fracked landscapes. Tornadoes through pipeline-riddled fields. Tsunamis across poisoned waters’ – all these disasters, clearly identified as the product of extractive capitalism, bring about mass death.^79^ Widespread sickness, both physical and mental, ensue. ‘The Earth was broken. Too much taking for too damn long’ and so are people:

the powers that be still refused to change and bent the already stooped under the whips of a schedule made for a population twice its size and inflated by the need to rebuild. Those that were left worked longer, worked harder…. And so they got sicker, this time in the head. They stopped dreaming.^80^

The sleep crisis of the settler population originates in the trauma of environmental collapse and, at the same time, in the colossal amount of work – both actual work and of repression – required to continue sustaining extractive capitalism’s unsustainable pace and voraciousness, even in the face of its evident failure. There are structural parallels, Dimaline’s series suggests, between the impossible demands of a 24/7 system on the planet and on our bodies and minds.

The imagery of machines pervades Dimaline’s descriptions of the dreamless, echoing Crary’s point that 24/7 is a ‘model of machinic performance and a suspension of living’.^81^ ‘[A] man without dreams is just a meaty machine with a broken gauge’, so much so that French, the seventeen-year-old protagonist of the series, imagines that when the dreamless die they ‘just shu[t] off like factory machines at the end of a shift: functioning, purposeful, and then just out’.^82^ Dreams, as Huebener notes, operate both literally and figuratively in the series. On the one hand, ‘REM sleep and dreams are vital for sustaining emotional and mental health’, which is why the dreamless lose their minds and kill themselves and others. On the other hand, as we have begun to see with *Sleep Donation*, dreams convey ‘the capacity to foster hope, meaning, purpose and vision for the future’.^83^ Without this capacity, settlers are reduced to their mere function as cogs in the proverbial capitalist machinery. Yet, the sleep crisis of the settler population does pose a threat to this system, as the dreamless can get so depressed that they stop fulfilling their machinelike functions and ‘refus[e] to work at all’, which is ‘even worse for the new order’ than their deaths.^84^

Indeed, in *The Marrow Thieves* and *Hunting by Stars*, despite the environmental collapse, extractive capitalism is hell-bent on perpetuating itself for as long as possible, which also perpetuates colonial oppression. This project is inherently doomed – that the settlers have lost their ability to reproduce further emphasises the system’s lack of futurity^85^ – and thus works to remove all ‘dreams’ of possible alternatives. Indigenous people can still dream, embodying hope for futures beyond extractive capitalism and its environmental destruction. Hardly by chance, as Rose – one of the Indigenous survivors the series revolves around – explains, Indigenous dreams are about their lands: ‘My dreams are full of lakes and the small islands that skip across them like a heartbeat. They are all that I am. They are *my* land … Our lands are who we are’.^86^ It is this hope for alternative futures that extractive capitalism seeks to squash by returning to the past of the Residential Schools system.^87^ Through the extraction of bone marrow, the radical potential of Indigenous dreams is erased, as the dreamers are turned into ‘fuel’ for the machines that are the settlers and left to die once their dream-resources are exhausted.^88^ As in *Sleep Donation*, sleep becomes a resource for extractive capitalism. Unlike Russell’s novel, though, in *The Marrow Thieves* and *Hunting by Stars* it is Indigenous sleep specifically that is exploited and exhausted to keep the system going. Tellingly, both Russell and Dimaline deploy the image of babies to convey the horrors of the extraction and commodification of sleep. But where in *Sleep Donation*, Baby A is a resource whose safety the Slumber Corps seem to want to protect, at least in principle, no such concerns exist for Dimaline’s future Canadian Government, who are have decided to ‘focus on farming [Indigenous] newborns’ as their stem cells are easier to extract.^89^

The theft of Indigenous sleep and the disposability of Indigenous dreamers draws attention to extractive capitalism’s racial inequalities and violent dispossessions. As Françoise Vergès underlines, the politics of extraction and exhaustion are racialised: ‘to accumulate wealth, the capitalist economy mines the life force of people of colour around the world until their deaths’.^90^ While these dynamics have their origin in colonisation and the slave trade, they also continue to this day. It is upon an invisible ‘economy of exhaustion’ of precarious and endangered people of colour, especially women of colour who work in the cleaning and caring sectors, that the rest of the economy is based.^91^ Lack of sleep is, therefore, not just a matter of public health, as the discourse of the sleep crisis suggests, but of social and racial justice.^92^

Dimaline situates the bone marrow extraction in a long history of commodification and plundering of Indigenous resources, lives, and labour, highlighting the shared colonial roots of the economy of exhaustion and the environmental crisis.^93^ The ‘Story’ rituals, through which Miig, the leader of the group of Indigenous survivors that become French’s family, keeps alive the group’s historical memory, trace this long history. Story starts with the violence of the first encounters with the colonisers, continues with the original Residential Schools, where Indigenous peoples ‘almost lost [their] languages’, then turns to the ‘wars for the water’, when ‘America reached up and started sipping on [Indigenous] lakes with a great metal straw’, ‘pollut[ing] [them] to muck’, and finally to the ‘new world’ of widespread environmental collapse, where settlers stop dreaming.^94^ As more and more of North America becomes uninhabitable due to toxic spills, melting glaciers and rising water levels, Indigenous peoples first become, once again, the target of land dispossession. They are forcibly removed from lands that are appropriated by companies determined to carry on with their indiscriminate extractive practices notwithstanding the devastation these have already caused.^95^ Then, it is Indigenous peoples’ very bodies that become ‘supply’ for extractive capitalism to exploit and exhaust.^96^ Indigenous peoples, in other words, bear the brunt of both climate breakdown and the sleep-apocalypse. Through the close links Story develops between the extraction of resources from Indigenous lands and bodies, we can see colonialism’s ‘parallel construction of “nature” as resource and the dehumanization of Indigenous peoples’ in action.^97^ It is not just that sleep is aligned with nature here, but that Indigenous sleepers are dehumanised as natural resources and made disposable.

*The Marrow Thieves* and *Hunting by Stars* articulate an important corrective to the sleep crisis discourse we have seen articulated in texts so far. As Reiss points out,

our society is undergoing two sleep crises: a psychological one in which those who live in relative states of comfort try to wrestle their sleep into submission, and a more existential struggle experienced by those who are expected to sleep by the rules of others yet are denied the time, space and security to do so.^98^

Focus on the former, however, tends to obscure the latter. Through the literal exhaustion of Indigenous sleepers, Dimaline’s series brings the latter sleep crisis to the fore, suggesting that, just as the effects of climate change are not evenly distributed but disproportionately fall on the Global South and Indigenous communities, the bodily exhaustion produced by extractive capitalism is most intensively felt by these same communities.

## Sleep against extractive capitalism and climate breakdown

Since extractive capitalism is imbricated with both bodily and planetary exhaustion, contemporary texts like Finegan Kruckemeyer’s play *Hibernation* and Tega Brain and Sam Lavigne’s Perfect Sleep app conceive of further rest as a behavioural change that may help us address not just the sense of a sleep crisis but also the threat of climate breakdown by forcing systemic change. *Hibernation* takes place in the 2030s, a time when the world is on the brink of environmental collapse. There have been continuous mass food shortages and nine nations are under water.^99^ Governments are fixated on space colonisation as the only possible solution to this crisis but the research effort behind the space race gives Emily Metcalfe, an Australian government aide, a much simpler solution. Rather than using ‘54E’, a ‘physical suppressant [that] slows metabolism [and] halts your body’s needs’ to facilitate the migration to Mars, Emily urges the Minister for Space Exploration Grant Warwick to deploy the drug to hibernate the world’s human population for a year.^100^ Echoing the environmentalist slogan ‘there is no planet B’, Emily explains: ‘I think I’ve found a planet which will satisfy all our needs’ and ‘instead of doing *everything* we can to reach it, I propose … we do *nothing* we can’, that is, go to bed and sleep the environmental crisis off in the hope that, in the total absence of human activity, the Earth will regenerate.^101^ Hiber-nation and its complete shut-down of extractivism have their desired effects: the number of birds increases exponentially, coral reefs are back, oceans once again teem with fish, and Bangladesh has clean water for another decade. Thus, Hibernation soon becomes a popular movement calling for a year of suspension of the capitalist machine every ten, and the play ends just as the second Hibernation starts.

Hibernation’s success is not without issues, however. The imaginary of Covid looms large over the play, with the pandemic being an inspiration for Emily’s vision:

there were these reports coming in from Wuhan – from the first 25 days where they shut all its factories. And mostly they just talk about the money loss. *But alongside that* are these NASA images showing carbon dioxide levels *dropping*. … And that’s 25 days. With *365*… Anything could happen.^102^

Indeed, the ‘nature is healing, we are the virus’ social media posts that went viral during the Covid pandemic, were later debunked and parodied through memes are another – albeit not explicitly mentioned^103^ – influence on Emily’s proposal, suggesting eco-fascism as a lens to interpret the problematic elements of the Hibernation movement.

The first Hibernation was not a unanimous decision. Seven countries were against it, yet once in the atmosphere, the 54E gas affected the world’s entire human population, irrespective of governments’ and individual people’s preferences. To a journalist’s objection that, by not having an opt-out clause, Hibernation is effectively a dictatorship that does away with ‘some pretty big human rules’, Emily retorts: ‘*Human rules* are the reason we’re in this shit! We have *ruled* over every species. We have *ruled* over the planet. We’ve *ruled* over each other. And everyone is hurting *because* of those rules’.^104^ The ‘we’ Emily uses here echoes the ‘we’ of the ‘we are the virus’ Covid posts, whose eco-fascism ‘collapses the species into an undifferentiated biological mass taken to be essentially destructive of the planet’s ecology … ultimately concluding that some portion of humanity ought to be exterminated’.^105^ This conceptual collapse obscures the profound inequalities that characterise the environmental crisis, both in terms of the populations responsible for the highest emissions now and historically – the Global North – and in terms of the populations bearing the brunt of the emergency – the Global South and Indigenous peoples. These inequalities continue to underlie Hibernation. As Chidera, whose son drowned because Hibernation ‘turned [Lagos] into rivers’, puts it: ‘The *rules for Hibernation* were made in Washington, in Shangai. In Berlin.. But *the price for Hibernation* – it was paid in Lagos’ – notice how Chidera’s words echo Emily’s about rules, subverting their homogenisation by highlighting the unequal effects of these rules.^106^

Yet, beyond the hyperbolic and eco-fascist nature of the Hibernation proposal, lies the more fundamental suggestion that both the human and more-than-human worlds need rest from continuous extraction. Hardly by chance, Emily’s idea for Hibernation is born out of a sleep crisis of sorts, for she is ‘so tired of being so tired, of losing sleep worrying what’ll happen to us’ that during one of her many sleepless nights she realises that sleep itself could be an answer to the world’s environmental predicament.^107^ This answer is also explored by the Perfect Sleep app, which is based on the premise that ‘lack of sleep and climate change are both products of the same extractivist capitalist system where regeneration, rest and natural limits go unvalued’.^108^ The sleep schedule and alarm functionality of the Perfect Sleep app aim at decreasing worktime and productivity by steadily increasing time spent asleep until the sleeper eventually achieves total sleep. This impossible goal humorously subverts the language of goals and achievements that characterises today’s ubiquitous sleep-tracking apps and devices, which entrench extractive capitalism by upholding an instrumental view of rest as merely a tool for productivity.^109^ Indeed, echoing the role of dreams as spaces of resistance discussed through Dimaline’s series, the app’s dream incubation soundscapes ‘invite sleepers to dedicate their dreamspace to envisaging a world beyond our own’ and the present system.^110^ Sleep and dreams are therefore conceived as ‘a potential climate engineering technology’, as Brain and Lavigne model how increased time for rest would ultimately drive emissions down.^111^

Human hibernation is of course not an option, but Kruckemeyer’s as well as Brain and Lavigne’s provocations chime with a wider sense that the current system is unsustainable for both the environment and people’s lives and that we should reassess how today’s society values sleep and rest. Hardly by chance, less hyperbolic ideas than total sleep routinely go viral on social media. Consider, for instance, the call by environmental historian Jayson M. Porter for ‘investing in more sleep for climate justice’, or the ‘rest is resistance’ framework created by Tricia Hersey, also known as The Nap Ministry, who posits rest as a way to resist grind culture and white supremacy, and to repair the Earth from the disastrous environmental effects produced by grind culture’s push for constant production and consumption.^112^ To return to Fraser’s boundary struggles, by pushing back against capitalism’s pervasive extraction and exhaustion of resources – bodily and planetary – further sleep may not just help us address the struggle over the boundary between the time for production and the time for social reproduction but the struggle over the boundary between human society and non-human nature.

The politics of sleep and the politics of the environment appear, therefore, to be closely intertwined in the twenty-first century. By exploring eco-anxiety as well as by conjuring up pandemics of sleep disorders or states of total sleep, the texts discussed in this article highlight how, when it comes to the climate crisis, the interrogation of the systems producing the crisis should go hand in hand with the interrogation of the health impacts these systems have on our bodies and minds. Through its boundary position between the individual and the systemic, sleep offers an important vantage point to do just so, drawing attention to structural parallels between human and environmental exhaustion. And, because of extractive capitalism’s similar devaluation of sleep and nature, the re-evaluation of sleep and rest also offers a pathway to begin working towards a world where human and planetary health are more substantially fostered. To put it with Crary’s evocative words, ‘the imaginings of a future without capitalism’ and, I would add, beyond climate collapse, may indeed ‘begin as dreams of sleep’.^113^

## Notes

1 Met Office Press Office, ‘Summer 2022: A Historic Season for Northern Hemisphere Heatwaves’, 29 July 2022, https://blog.metoffice.gov.uk/2022/07/19/summer-2022-a-historic-season-for-northern-hemisphere-heatwaves/ [Date accessed: 1 September 2022]. At the time of revising this article for publication, 2023 is on track for becoming ‘the world’s hottest year’; Justin Rowlatt, ‘Excessive Heat: Why This Summer Has Been So Hot’, *BBC*, 13 July 2023, https://www.bbc.co.uk/news/science-environment-66143682 [Date accessed: 14 July 2023].

2 Benji Jones, ‘This Could Be the Coolest Summer of the Rest of Your Life’, Vox, 22 June 2022, https://www.vox.com/energy-and-environment/2022/6/22/23176860/heat-wave-summer-temperatures-climate-change-us-europe [Date accessed: 1 September 2022]; Met Office Press Office, ‘Climate Study Backs Up Met Office Research’, 29 July 2022, https://blog.metoffice.gov.uk/2022/07/29/climate-study-backs-up-met-office-research/ [Date accessed: 1 September 2022].

3 See, for instance, Benjamin Lynch and Christian Abbott, ‘How to Sleep in a Heatwave as UK Battles Record-Breaking 40C Temperatures’, *Mirror*, 19 July 2022, https://www.mirror.co.uk/news/health/how-sleep-heatwave-uk-bakes-27254206 [Date accessed: 1 September 2022]; Sarah Young and Kate Ng, ‘How to Sleep during a Heatwave, According to Experts’, *Independent*, 24 August 2022, https://www.independent.co.uk/life-style/health-and-families/how-to-sleep-uk-heatwave-2022-b2142228.html [Date accessed: 1 September 2022]. As we shall see shortly, media pieces outlining top tips for a good night sleep are evergreen components of the discourse of the so-called sleep crisis.

4 ‘At every “node” along [the extractive] circuit’, writes Ajay Singh Chaudhary, ‘“inputs” – ecological, political, social, individual – are extracted and “exhausted”‘ in ‘one of the most vicious cycles imaginable’, for ‘capital must keep burning through more of the biosphere and the human systems inside it to keep up profit margins’; ‘The Extractive Circuit’, *The Baffler*, no. 60, November 2021, https://thebaffler.com/salvos/the-extractive-circuit-singh-chaudhary [Date accessed: 14 July 2022].

5 Lancet, ‘A Commission on Climate Change’, *The Lancet*, 373.9676 (2009), p. 1659; Alistair Woodward et al., ‘Climate Change and Health: On the Latest IPCC Report’, *The Lancet*, 383.9924 (2014), pp. 1185–89.

6 Nick Obradovich et al., ‘Nighttime Temperature and Human Sleep Loss in a Changing Climate’, *Science Advances*, 3.5 (2017), n.p; Kelton Minor et al., ‘Rising Temperatures Erode Human Sleep Globally’, *One Earth*, 5.5 (2022), pp. 534–49.

7 Steven W. Lockley and Russell G. Foster, *Sleep: A Very Short Introduction* (Oxford: Oxford University Press, 2011).

8 Helen Louise Berry et al., ‘Climate Change and Mental Health: A Causal Pathway Framework’, *International Journal of Public Health*, 55.2 (2010), pp. 123–32.

9 American Psychological Association, ‘Mental Health and Our Changing Climate: Impacts, Implications, and Guidance’, March 2017, https://www.apa.org/news/press/releases/2017/03/mental-health-climate.pdf [Date accessed: 14 July 2022], p. 29.

10 Chiara Baglioni et al., ‘Sleep and Mental Disorders: A Meta-Analysis of Poly-somnographic Research’, *Psychological Bulletin*, 142.9 (2016), pp. 969–90.

11 American Psychological Association, ‘Mental Health’.

12 Till Roenneberg, ‘The Human Sleep Project’, *Nature*, 498 (2013), pp. 427–28, p. 427.

13 Gautam Rao et al. ‘Informing Sleep Policy Through Field Experiments’, *Science*, 374.6567 (2021), pp. 530–33, p. 530.

14 Lockley and Foster, *Sleep*, p. 3.

15 Jim Horne, *Sleeplessness: Assessing Sleep Need in Society Today* (Basingstoke: Palgrave, 2016), p. 38. For another perspective cautious of the sleep crisis thesis, see Simon J. Williams, *Sleep and Society: Sociological Ventures into the (Un)Known* (London: Routledge, 2005); Simon J. Williams, *The Politics of Sleep: Governing (Un)consciousness in the Late Modern Age* (Basingstoke: Palgrave, 2011).

16 Cf. for instance Miranda Larbi, ‘NIGHTMARE: Getting less than seven hours’ sleep a night could KILL you as experts warn Brits are in the grip of a “sleep crisis”‘, *The Sun*, 12 July 2019, https://www.thesun.ie/news/4320563/getting-less-than-seven-hours-sleep-will-kill-you-experts-warn-brits-are-in-the-grip-of-a-sleep-crisis/ [Date accessed: 14 July 2022]; Anna North, ‘The Pandemic Has Created a Nation of Insomniacs’, *Vox*, 16 Sept. 2021, https://www.vox.com/22671566/sleep-deprived-insomnia-covid-pandemic-stress-anxiety [Date accessed: 14 July 2022].

17 Diletta De Cristofaro and Simona Chiodo, ‘Quantified Sleep: Self-Tracking Technologies and the Reshaping of 21st-Century Subjectivity’, *Historical Social Research*, 48.2 (2023), pp. 176–93; Diletta De Cristofaro, ‘Insomnia’, in Robert Meadows and Christiane Solte-Gresser (eds), *A Cultural History of Sleep and Dreaming in the Age of Globalization* (London: Bloomsbury, forthcoming).

18 See, for instance, Lee Scrivner, *Becoming Insomniac: How Sleepless Alarmed Modernity* (Basingstoke: Palgrave, 2014); Anna Katharina Schaffner, *Exhaustion: A History* (New York: Columbia University Press, 2016); Sally Shuttleworth, ‘Fagged Out: Overwork and Sleeplessness in Victorian Professional Life’, *Interface Focus*, 10.3 (2020).

19 Schaffner, *Exhaustion*, p. 242.

20 Cf. Chaudhary, ‘The Extractive Circuit’: ‘Feelings of exhaustion – depression, desperation, fatigue, exasperation – course through [the extractive circuit’s] wirings, neurons, biochemicals, and sinews’. Consider, in this sense, the popularity of the notion of burnout, the subject of countless viral essays, such as Anne Helen Petersen’s ‘How Millennials Became the Burnout Generation’ (*Buzzfeed*, 5 January 2019, https://www.buzzfeednews.com/article/annehelenpetersen/millennials-burnout-generation-debt-work [Date accessed 16 August 2022]), which she later turned into a book. For academic perspectives on today’s ‘burnout society’, see Byung-Chul Han, *The Burnout Society* (Stanford CA: Stanford University Press, 2015); as well as Sighard Neckel, Anna Katharina Schaffner, and Greta Wagner (eds), *Burnout, Fatigue, Exhaustion: An Interdisciplinary Perspective on a Modern Affliction* (Cham: Palgrave, 2017).

21 Benjamin Reiss, ‘Sleep’s Hidden Histories’, *Los Angeles Review of Books*, 15 Feb. 2014, https://lareviewofbooks.org/article/sleeps-hidden-histories/ [Date accessed: 24 July 2022]. The moniker of ‘critical sleep studies’ comes from this article, and has since been widely adopted by scholars – cf., for instance, Michael Greaney, *Sleep and the Novel: Fictions of Somnolence from Jane Austen to the Present* (Basingstoke: Palgrave, 2018); Paul Huebener, ‘Stealing Sleep: Expanding the Conversation on the Literary Politics of Sleep and Insomnia’, *English Studies in Canada*, 44.3 (2018), pp. 67–89. For a survey of recent work in the field, see Simon J. Williams and Matthew Wolf-Meyer, ‘Longing for Sleep: Assessing the Place of Sleep in the 21st Century’, *Somatosphere*, March 2013, http://somatosphere.net/series/longing-for-sleep/ [Date accessed: 24 July 2022].

22 Williams, *Sleep and Society*, p. 104.

23 I am following Williams’ point that, qua ‘prism’, sleep provides a ‘novel vantage point from which to explore any or all other aspects of social, cultural, historical and political life’; Williams and Matthew Wolf-Meyer, ‘Longing for Sleep’.

24 See, for instance, Jonathan Crary, *24/7: Late Capitalism and the Ends of Sleep* (London: Verso, 2013); Matthew Wolf-Meyer, *The Slumbering Masses: Sleep, Medicine, and Modern American Life* (Minneapolis: University of Minnesota Press, 2012); Williams, *Politics*.

25 As Huebener writes, ‘The fields of critical sleep studies and literary studies have only just begun to cross paths, and developing the interactions between these approaches promises to bring about startling insights’; ‘Stealing Sleep’, p. 68.

26 Jonathan Crary’s *24/7* and Benjamin Reiss’ *Wild Nights: How Taming Sleep Created Our Restless World* (New York: Basic Books, 2017) are particularly generative. The growing scholarly interest in the relationship between the politics of sleep and the politics of the environment in the twenty-first century is evident in two events organised in 2023 by The Sociability of Sleep researchcreation project (see https://sociabilityofsleep.ca/ [Date accessed 15 July 2023]), ‘Hot Takes’, a World Sleep Day roundtable on sleep and the climate crisis (available at https://youtu.be/gzFiEud597Y [Date accessed 15 July 2023]), and ‘Sleep Salon 14: Sleeping in Time’, where Huebener asked provocative questions that resonate with my analyses in this article: ‘How can we learn to invent and share good sleep amidst the anxieties of time running out [because of the climate crisis]?. How can we learn to measure climate justice in part through equitable access to good sleep?’ (available at https://youtu.be/uY8Vno6VlMg [Date accessed 15 July 2023]).

27 See, for instance, Wai Chee Dimock, ‘Endangered’, *PMLA*, 134.2 (2019), pp. 233–41; Jonathan Coope, ‘On the Need for an Ecologically Dimensioned Medical Humanities’, *Medical Humanities*, 47.1 (2021), pp. 123–27.

28 Rithika Ramamurthy, ‘Personal Hell: The Climate Anxiety Novel’, *The Drift*, 6 May 2021, https://www.thedriftmag.com/climate-anxiety/ [Date accessed 1 September 2022]; Parul Sehgal, ‘How To Write Fiction When the Planet is Falling Apart’, *The New York Times Magazine*, 5 Feb. 2020, https://www.nytimes.com/2020/02/05/magazine/jenny-offill-weather-book.html [Date accessed 1 September 2022].

29 Jenny, Offill, *Weather* (London: Granta, 2020), p. 10.

30 Ibid., pp. 83, 76.

31 Ibid., p. 85.

32 Ibid.

33 Ibid., p. 89.

34 Ibid., p. 106.

35 Ibid., p. 178.

36 Ibid.

37 Ibid., p. 187.

38 Ramamurthy, ‘Personal Hell’.

39 Ibid.

40 Ibid.

41 Timothy Morton, *The Ecological Thought* (Cambridge, MA: Harvard University Press, 2010), p. 28.

42 Clare Fisher, ‘The Centrality of the Trivial’, *Alluvium*, 8.2 (2020), n.p.’

43 Sehgal, ‘How To Write’.

44 Williams, *Politics*, p. x.

45 Nancy Fraser and Rahel Jaeggi, *Capitalism: A Conversation in Critical Theory* (Cambridge: Polity, 2018), p. 118.

46 Offill, *Weather*, p. 123.

47 Ibid., p. 44.

48 Williams, *Politics*, p. xviii.

49 Offill, *Weather*, p. 20.

50 Ibid., p. 123.

51 Crary, *24/7*, pp. 17, 10.

52 Fraser and Jaeggi, *Capitalism*, p. 118.

53 Crary, *24/7*, p. 24.

54 Offill, *Weather*, p. 67.

55 https://www.obligatorynoteofhope.com/ [Date accessed 1 September 2022]. The website consists of forty-five ‘tips for trying times’ and links to activist organisations.

56 Ibid., p. 201.

57 Beyond the novels discussed in this article, examples of sleep-apocalypse fiction include Charlie Houston’s *Sleepless* (2010), Adrian Barnes’ *Nod* (2012), Kenneth Kalhoun’s *Black Moon* (2014), Julia Armfield’s ‘The Great Awake’ (2018), H.G. Bells’ *Sleep Over: An Oral History of the Apocalypse* (2018), and Karen Thompson Walker’s *The Dreamers* (2019). For another analysis of this body of texts, see Alice Bennett’s article, which also reads the emergence of these fictions alongside the sleep crisis discourse; ‘“People with Equal but Opposite Afflictions, Propping Each Other Up”: Sleep Solidarity and Fictions of Mass Sleeplessness’, *Modern Fiction Studies*, 68.3 (2022), pp. 525–43. Bennett focuses specifically on novels about mass sleeplessness and our respective arguments illuminate different, albeit complementary, aspects of the genre, with her article tracing how the narratives in question ‘identify the inequalities that underpin both the sleep crisis of late capitalism’s always-on, technologically augmented colonisation of rest time and the slow, persistent exhaustion of those who are homeless, displaced, or lacking healthcare’ (Ibid., p. 541). Cf. also the conclusion of Greaney’s *Sleep and the Novel*, which dedicates a few pages to the ‘post-dormative novel’. This companion to the ‘sleep crisis narrative’, Greaney maintains, imagines ‘sleep’s imminent abolition in order to cure us of our habitual over-valuation of wakefulness’ (Greaney, *Sleep and the Novel*, pp. 212–13), a claim that resonates with my argument that the genre’s environmental thread aims at highlighting the costs of this over-valuation not just for human health but for planetary health.

58 Karen Russell, *Sleep Donation* (New York: Vintage, 2020), p. 14.

59 Ibid., p. 15.

60 Bennett, ‘Sleep Solidarity’, p. 532.

61 Russell, *Sleep Donation*, p. 15.

62 Amanda Stern, ‘Karen Russell: Sleep Donation’, *Bookable*, 27 October 2020, https://podcasts.apple.com/us/podcast/karen-russell-sleep-donation/ id1493225830?i=1000496200862 [Date accessed: 1 October 2022].

63 Russell, *Sleep Donation*, p. 16; emphasis mine.

64 Ibid., p. 15-16.

65 Val Plumwood, ‘Decolonizing Relationships with Nature’, in William M. Adams and Martin Mulligan (eds), *Decolonizing Nature: Strategies of Conservation in a Postcolonial Era* (London: Earthscan, 2003) pp. 51–78, p. 52. Cf. also Reiss’ argument that ‘our sense that we can *conquer* sleep, tame it, make it conform, relies on [an] environmentally devastating mindset. : an attitude of dominion over nature (including our own bodies) through technology and consumerism’; *Wild Nights*, pp. 14–15; emphasis in original.

66 A well-known shift in this sense is the move from pre-industrial times’ segmented sleep (that is, sleep that takes place in segments in the night, as well as naps during the day) to consolidated sleep (that is, sleep that takes place in one block during the night), which served the interests of factories’ consolidated work-days; Roger A. Ekirch, *At Day’s Close: Night in Times Past* (New York: W. W. Norton, 2006); Wolf-Meyer, *Slumbering Masses*.

67 Crary, *24/7*, pp. 9–10.

68 Stern, ‘Karen Russell’. Consider also the parallels with Greta Thunberg’s famous speech at the U.N. 2019 Climate Action Summit, where the activist accused world leaders of stealing her dreams with their inaction. NPR, ‘Transcript: Greta Thunberg’s Speech at The U.N. Climate Action Summit’, *NPR*, 23 Sept. 2019, https://www.npr.org/2019/09/23/763452863/transcript-greta-thunbergs-speech-at-the-u-n-climate-action-summit [Date accessed: 1 October 2022].

69 Russell, *Sleep Donation*, p. 129.

70 Ibid.

71 Ibid., p. 92.

72 Cf. Sharae Deckard, who illustrates how *Sleep Donation* is pervaded throughout by a ‘language of exhaustion. overlaid with an imaginary of intensified water and oil extraction’; ‘“Terminal Insomnia”: Sleeplessness, Labor, and Neoliberal Ecology in Karen Russell’s *Sleep Donation* and Alex Rivera’s *Sleep Dealer*’, in Liam Kennedy and Stephen Shapiro (eds), *Neoliberalism and Contemporary American Literature* (Lebanon, NH: Dartmouth College Press, 2019), pp. 187–206, p. 196.

73 Williams, *Politics*, p. xviii.

74 See for instance Marco Hafner et al., ‘Why Sleep Matters – The Economic Cost of Insufficient Sleep: A Cross-Country Comparative Analysis’, *RAND Health Quarterly*, 6.4 (2017), n.p.

75 Crary, *24/7*, p. 10.

76 Deckard, ‘Terminal Insomnia’, p. 189.

77 Russell, *Sleep Donation*, p. 81.

78 Ibid., p. 82.

79 Cherie Dimaline, *Hunting by Stars* (New York: Abrams, 2021), p. 22.

80 Cherie Dimaline, *The Marrow Thieves* (London: Jacaranda, 2019), pp. 100–01.

81 Crary, *24/7*, p. 9.

82 Dimaline, *Marrow*, pp. 101, 25.

83 Huebener, ‘Stealing Sleep’, p. 81-82.

84 Dimaline, *Marrow*, p. 101.

85 Dimaline, *Hunting*, p. 154.

86 Ibid., p. 160; emphasis in original.

87 As Huebener writes, by ‘leaving behind dead cultures. the residential school system was always in the business of stealing dreams’; ‘Stealing Sleep’, p. 84.

88 Dimaline, *Hunting*, p. 5.

89 Ibid., p. 288.

90 Françoise Vergés, ‘On the Politics of Extraction, Exhaustion and Suffocation’, *Internationale Online*, 8 Nov. 2021, https://www.internationaleonline.org/research/politics_of_life_and_death/195_on_the_politics_of_extraction_exhaustion_and_suffocation/ [Date accessed: 14 October 2022].

91 Ibid.

92 Cf. also Jonathan White, ‘Circadian Justice’, *The Journal of Political Philosophy*, 30.4 (2022), pp. 487–511.

93 I develop this argument in terms of the colonial origins of the Anthropocene in Diletta De Cristofaro, ‘Patterns of Repetition: Colonialism, Capitalism and Climate Breakdown in Contemporary Post-Apocalyptic Fiction’, *Parallax*, 27.1 (2021), pp. 12–30.

94 Dimaline, *Marrow*, pp. 34–37.

95 Ibid., pp. 35, 102.

96 Dimaline, *Hunting*, p. 72.

97 Moritz Ingwersen, ‘Reclaiming Fossil Ghosts: Indigenous Resistance to Resource Extraction in Works by Warren Cariou, Cherie Dimaline, and Nathan Adler’, *Canadian Literature*, 240 (2020), pp. 59–76.

98 Reiss, *Wild Nights*, p. 7.

99 Finegan Kruckemeyer, *Hibernation*, PDF courtesy of the playwright (2021), p. 9. I would like to express my gratitude to Finegan for sharing the script of *Hibernation* with me.

100 Ibid., p. 14.

101 Ibid., pp. 14, 7; emphasis and ellipses in original.

102 Ibid., p. 15; emphasis and ellipses in original.

103 Kai Bosworth, ‘The Bad Environmentalism of “Nature is Healing” Memes’, *Cultural Geographies*, 29.3 (2022), pp. 353–74. Kruckemeyer does evoke these posts by describing post-Hibernation Earth as Venice (*Hibernation*, p. 49), whose canals, according to the later debunked posts, saw the return of swans and dolphins during Covid.

104 Kruckemeyer, *Hibernation*, p. 22; emphasis in original.

105 Bosworth, ‘Bad Environmentalism’, p. 369.

106 Kruckemeyer, *Hibernation*, pp. 60–61; emphasis in original.

107 Ibid., p. 15.

108 Tega Brain and Sam Lavigne, ‘Perfect Sleep’, 2021, https://perfectsleep.labr.io/ [Date accessed: 1 November 2022].

109 De Cristofaro and Chiodo, ‘Quantified Sleep’.

110 Brain and Lavigne, ‘Perfect Sleep’.

111 Ibid.

112 @RogueChieftan, ‘I’m still stuck on this idea: investing in more sleep for climate justice. If everyone in the United States was encouraged to sleep an additional hour or two, the reduction in greenhouse gas emissions could be huge. Like the size of smaller, more sustainable countries huge.’ *Twitter*, 1 March 2022, 4:19pm, https://twitter.com/RogueChieftan/status/1498694446766080004 [Date accessed 3 November 2022]; @TheNapMinistry, *Twitter*, https://twitter.com/TheNapMinistry [Date accessed 3 November 2022].

113 Crary, 24/7, p. 128.

